# Cartas al editor

**Published:** 2020-03-30

**Authors:** Marisol Angulo-Ramos, Perú César Merino-Soto

**Affiliations:** 1 Universidad Católica Los Ángeles de Chimbote, Universidad Católica de Los Ángeles de Chimbote Universidad Católica Los Ángeles de Chimbote Peru; 2 Universidad de San Martín de Porres, Perú Universidad de San Martín de Porres Universidad de San Martín de Porres Peru

Cuernavaca, 26 de noviembre de 2019

Señores Comité Editorial Revista *Biomédica* Bogotá

Estimados señores:

Hace poco, en el estudio observacional de Lopera y Lemos "Factores socioeconómicos y clínicos asociados con infecciones oportunistas en pacientes con HIV afiliados al sistema de salud", aparecido en el número 39 de *Biomédica*^1^, se hizo un exhaustivo análisis y se presentó la prevalencia de infecciones oportunistas asociadas con factores clínicos y socioeconómicos. Dada su importancia para documentar posibles decisiones de salud pública, en esta carta resaltamos dos aspectos analíticos sobre dicha prevalencia según los grupos de edad (reportada en el cuadro 3 del citado artículo), que requieren atención para interpretar correctamente los resultados.

En primer lugar, el informe de esta relación únicamente presentó el valor de p asociado con el rechazo de la hipótesis nula cuando, en su lugar, la información del tamaño o de la fuerza de la asociación arrojaría una mejor descripción. En segundo lugar, tal como lo señalan los autores, se puede observar una posible tendencia monotónica ascendente de la prevalencia de las infecciones oportunistas en relación con los grupos de edad. Esta asociación fue aparentemente evaluada mediante la prueba de ji al cuadrado para categorías no ordenadas, la cual tiende a incrementar el error de tipo II cuando las categorías están realmente ordenadas (esto es, los grupos de edad) ^2^. Para ahondar más, la forma del patrón ascendente parece lineal y en otras este patrón aparentemente no lo es. Sin embargo, estas observaciones son esencialmente subjetivas y deben ser probadas directamente con los datos presentados en el cuadro 3.

Para complementar esta ausencia, se aplicó una prueba de tendencia lineal en la distribución de las frecuencias ^3^^,^^4^ del cuadro 3, que consiste en determinar si un ordenamiento lineal de las frecuencias (hipótesis alternativa) se da más allá del error de muestreo (hipótesis nula). Usualmente, se reportan el estadístico de x^2^ total, el x^2^ derivado de la tendencia lineal y la diferencia entre ambos, esta última interpretada como el desvío de la linealidad ^2^. Como denominador, se mantuvo el número de pacientes con HIV. Los resultados de este análisis se presentan en el siguiente cuadro:

**Prueba de:** tendencias de prevalencias

	χ**2 total (gl=4)**	Cochran-Armitage - χ2 (gl=1)	Desvío de la linealidad χ2 (gl=3)	Conclusión
Presencia de infecciones oportunistas	371,45**	338,79**	32,66**	No lineal
*Mycobacterium tuberculosis*	77,11**	70,47**	6,64ns	Lineal
Candidiasis	141,26**	137,39**	3,88ns	Lineal
Toxoplasmosis cerebral	136,68**	72,99**	60,69**	No lineal
Diarreas	20,41**	15,01**	5,40ns	No lineal
Neumonía por *Pneumocystis jirovecii*	50,67**	34,83**	15,85**	No lineal
*Herpes simplex*	40,05**	39,26**	0,79ns	Lineal
Criptococosis	41,37**	26,88**	14,49**	No lineal
Neumonía bacteriana	85,08**	0,02ns	85,05**	No lineal
Citomegalovirus	30,69**	28,34**	2,35ns	Lineal
Otras infecciones oportunistas	7,45ns	4,97*	2,48ns	No lineal

** p<0,01; ns: estadísticamente no significativo

Hallamos un leve predominio de tendencias no lineales, lo que supone que el patrón ascendente de las condiciones oportunistas puede tomar formas curvilíneas que merecen atención y no pueden tratarse únicamente como tendencias lineales. Esto puede tener relevancia clínica, o plantear una pregunta de investigación para explorar los factores determinantes de estos tipos de tendencia.
