# Supplementary material for: Cartas al editor
Source: Biomedica. 2020 Mar 30;40(1):208–10. [Article in Spanish] (PMC7357391)
Supplement: Related texts

# Reply: Cartas al editor

**Authors:** Mónica María Lopera, Yesly Lemos

**Affiliations:** 1 Línea de Investigación en Sistemas de Salud, Grupo de Gestión y Políticas de Salud, Facultad Nacional de Salud Pública, Universidad de Antioquia, Medellín, Colombia Universidad de Antioquia Facultad Nacional de Salud Pública Universidad de Antioquia Medellín Colombia; 2 Semillero de Investigación SISALUD, Facultad Nacional de Salud Pública, Universidad de Antioquia, Medellín, Colombia Universidad de Antioquia Facultad Nacional de Salud Pública Universidad de Antioquia Medellín Colombia

Medellín, 29 de enero de 2020

Señores Comité Editorial Revista *Biomédica* Bogotá

Estimados señores:

En relación con la carta titulada: "Tendencia lineal o no lineal de prevalencias", agradecemos el interés de los doctores Marisol Angulo Ramos y César Merino Soto en torno a nuestro estudio "Factores socioeconómicos y clínicos asociados con infecciones oportunistas en pacientes con HIV afiliados al sistema de salud".

En cuanto a los valiosos comentarios sobre la fuerza de la asociación y la linealidad de la prevalencia de las infecciones oportunistas en pacientes con VIH, queremos hacer las siguientes precisiones.

En efecto, como lo señalan los lectores, en el cuadro 2, que muestra la prevalencia de infecciones oportunistas por sexo en pacientes con HIV afiliados al Sistema General de Seguridad Social en Salud, 2012, se presentó el valor de p asociado con el rechazo de la hipótesis nula, pero no con la fuerza de la asociación. En los cuadros 4 y 5 del artículo original, se presentó la razón de momios *(odds ratio)* crudo y ajustado, respectivamente. Esta medida permite entender la fuerza de la asociación de las infecciones oportunistas con el sexo. La razón de prevalencia se presenta en el cuadro 1, que aquí incluimos para dar claridad al lector. Obsérvese la significación para todas las infecciones oportunistas, excepto la de la neumonía bacteriana.

**Cuadro 1:** Razón de prevalencia de infecciones oportunistas por sexo en pacientes con HIV afiliados al Sistema General de Seguridad Social en Salud, 2012

		Total		Femenino		Masculino	P	RP	IC95%
	n	% (IC95%)	n	% (IC95%)	n	% (IC95%)	Ji2		
Pacientes con HIV	37.325		10.059		27.266				
Personas con infecciones oportunistas	6.662	17,9 (17,5-18,2)	1.369	13,6 (12,9-14,3)	5.293	19,4 (18,9-19,9)	<0,01	1,43	(1,35-1,50)
*Mycobacterium tuberculosis*	2.123	5,68 (5,45-5,92)	416	4,13 (3,75-4,54)	1.707	6,26 (5,97-6,55)	0,02	1,51	(1,37-1,68)
Candidiasis	1.391	3,72 (3,53-3,92)	274	2,72 (2,41-3,06)	1.117	4,09 (3,86-4,33)	0,01	1,5	(1,32-1,71)
Toxoplasmosis cerebral	1.360	3,64 (3,45-3,83)	279	2,77 (2,46-3,11)	1.081	3,96 (3,73-4,20)	0,01	1,43	(1,26-1,63)
Diarrea	914	2,44 (2,29-2,61)	198	1,96 (1,70-2,25)	716	2,62 (2,43-2,82)	0,01	1,33	(1,14-1,56)
Neumonía por *Pneumocystis jirovecii*	907	2,43 (2,27-2,59)	178	1,76 (1,52-2,04)	729	2,67 (2,48-2,87)	0,01	1,51	(1,29-1,77)
Herpes simplex	696	1,86 (1,72-2,00)	136	1,35 (1,13-1,59)	560	2,05 (1,88-2,22)	0,01	1,52	(1,26-1,83)
Criptococosis	471	1,26 (1,15-1,38)	66	0,65 (0,50-0,83)	405	1,48 (1,34-1,63)	0,01	2,26	(1,76-2,91)
Neumonía bacteriana	469	1,25 (1,14-1,37)	115	1,14 (0,94-1,37)	354	1,29 (1,16-1,43)	0.12	1,14	(0,92-1,40)
Citomegalovirus	344	0,92 (0,82-1,02)	52	0,51 (0,38-0,67)	292	1,07 (0,95-1,20)	<0,01	2,07	(1,55-2,76)
Otras infecciones oportunistas	573	1,5 (1,4-1,7)	96	1,0 (0,8-1,2)	477	1,8 (1,6-1,9)	<0,01	1,83	(1,48-2,27)

En cuanto a la tendencia descrita en la relación de la edad con la presencia de infecciones oportunistas, en el artículo original no se planteó una tendencia monotónica, pero sí se señaló al aumento de la tendencia en la prevalencia de infecciones oportunistas al incrementarse la edad (cuadro 3 y figura 1). Además, se aclaró que existían leves variaciones y se presentaron los datos correspondientes que así lo muestran (página 191). Los cuadros 4 y 5 presentan el gradiente, positivo mediante la razón de momios cruda y ajustada, con significación estadística a partir del grupo de los 40 años.

Los autores no consideran apropiado efectuar pruebas utilizando la edad como variable continua por razones clínicas y estadísticas. La importancia clínica de la asociación de las infecciones oportunistas con la edad debe leerse con cautela, puesto que hay condiciones clínicas, fisiológicas y epidemiológicas que pueden contribuir a dichas variaciones. En efecto, la gran prevalencia de la neumonía bacteriana y la diarrea encontrada en niños se ajusta a la epidemiología de la enfermedad ^1^. La ligera reducción de infecciones como la toxoplasmosis cerebral y la criptococosis en personas mayores de 50 años debe estudiarse en futuras investigaciones. Los rangos de edad seleccionados en el estudio representan grupos de interés clínico y tienen un gran potencial para el ajuste de las guías clínicas.

Incluso si fuere relevante clínicamente, la linealidad propuesta por los lectores mediante la incorporación de una prueba de ji al cuadrado de tendencia presenta dos inconvenientes de tipo estadístico: en primer lugar, solo se requiere incorporar este tipo de pruebas cuando la variable independiente es cuantitativa ^1^^,^^3^ y, como se indicó, los grupos de edad seleccionados corresponden a rangos de interés clínico. En segundo lugar, dicha prueba de ji al cuadrado de tendencia no resulta concluyente, como lo supone el modelo de regresión logística, que sí ajusta el análisis según factores de confusión y que fue presentado en el cuadro 5 del artículo original.

## References

[1] Rudan I, Boschi-Pinto C, Biloglav Z, Mulhollandd K, Campbell H (2008). Epidemiology and etiology of childhood pneumonia. Bulletin of the World Health Organization.

[2] Kleinbaum DG, Kupper LL, Chambless LE (1982). Logistic regression analysis of epidemiologic data: Theory and practice. Communications in Statistics-Theory and Methods.

[3] Hosmer DW, Lemeshow S, Sturdivant RX (2013). Applied logistic regression.
